# Long-term real-world data of ustekinumab in Crohn’s disease: the Stockholm ustekinumab study

**DOI:** 10.1177/17562848241242700

**Published:** 2024-04-23

**Authors:** Francesca Bello, Samer Muhsen, Haider Sabhan, Alexandra Borin, Fredrik Johansson, Charlotte Höög, Ole Forsberg, Christina Wennerström, Charlotte Söderman, Mikael Lördal, Sven Almer

**Affiliations:** Karolinska Institutet, Department of Medicine Solna, Karolinska University Hospital, Centre for Digestive Health, Department of Gastroenterology, Dermatovenereology and Rheumatology, Stockholm, Sweden; Division of Gastroenterology and Hepatology, Department of Medicine, Danderyd Hospital, Danderyd, Sweden; Gastroenterology Unit, Medical Department, St Göran’s Hospital, Stockholm, Sweden; Department of Medicine, South Hospital, Stockholm, Sweden; Medical Library at Danderyd Hospital, Danderyd, Sweden; Department of Upper Digestive Diseases, Karolinska University Hospital, Stockholm, Sweden Department of Medicine Huddinge, Karolinska Institutet, Stockholm, Sweden; Janssen-Cilag AB, Stockholm, Sweden; Janssen-Cilag AB, Stockholm, Sweden; Department of Medicine Solna, Karolinska Institutet, Stockholm, Sweden Gastroenterology Unit, Medical Department, St Göran’s Hospital, Stockholm, Sweden; Division of Gastroenterology and Hepatology, Department of Medicine, Danderyd Hospital, Danderyd, Sweden; Karolinska Institutet, Department of Medicine Solna, Karolinska University Hospital, Centre for Digestive Health, Department of Gastroenterology B4:09, Dermatovenereology and Rheumatology, SE-171 76 Stockholm, Sweden

**Keywords:** Crohn’s disease, long-term follow-up, real-world data, ustekinumab

## Abstract

**Background:**

Ustekinumab is used to treat inflammatory bowel disease mainly in patients failing anti-tumour necrosis factor (TNF)-agents.

**Objectives:**

To provide real-world data in unselected patients with Crohn’s disease (CD), treated with ustekinumab.

**Design:**

Longitudinal retrospective study at four hospitals in Stockholm, Sweden.

**Methods:**

Disease activity (Harvey–Bradshaw index and physician global assessment), laboratory parameters, endoscopic findings and drug persistence were assessed. Follow-up data were obtained in patients that stopped ustekinumab.

**Results:**

In total, 322 patients (median age 38 years, 48% women) were included. All had luminal disease and 22% also fistulizing disease. A total of 271 (84%) had failed ⩾1 and 148 (46%) ⩾2 anti-TNF drugs; 34% failed vedolizumab. At inclusion, 93% had active disease; 28% were on oral corticosteroids and 18% on thiopurines. The median follow-up on treatment was 13.5 months; overall 67% were followed at least 24 months. By intention to treat analysis, response rate at 3 and 12 months was 43% and 42%, respectively. Among patients with ongoing ustekinumab, 19% were in steroid-free remission at 3 months and 64% at 12 months. The median faecal calprotectin level decreased from 460 µg/g at baseline to 156 µg/g at 3 months and was 182 µg/g at 12 months. C-reactive protein remained stable at 4 mg/L whereas serum albumin increased slightly. About 31% of patients were withdrawn during the first 12 months, mainly due to persisting disease activity 21%, adverse events 5%, bowel surgery 0.6% or malignancy 0.3%. The overall persistence on ustekinumab was 88%, 51%, 34% and 20% at 3, 12, 24 and 36 months, respectively. Within 12 months following withdrawal of ustekinumab in 121 patients, 64% had active disease most of the time, 68% needed another biologic and 24% underwent surgery.

**Conclusion:**

Among difficult-to-treat patients with CD, ustekinumab was effective in the majority, with high drug persistence at 12 and 24 months in combination with a favourable safety profile.

## Plain language summary

Study of a new biologic treatment in patients with Crohn’s disease that have failed previous medications

Why was the study done? New medical treatments become available in routine healthcare after rigorous testing on selected groups of patients in what is called clinical trials. The benefits and possible adverse events then need to be assessed in larger groups of unselected patients to gain a more generalizable knowledge of merits and shortfalls. Therefore studies in real-world settings are vital to complement the experience gained from clinical trials and they can provide robust data and reveal previously undetected safety issues. What did the researchers do? The research team studied the effect of a new injectable biological treatment, ustekinumab, in a large group of Swedish patients with an inflammatory bowel disease, Crohn’s disease, since the drug became available in routine health care. All patients had previously over long time periods tried several different treatments without obtaining stable symptom control. Information on the severity of disease, symptoms, laboratory tests, examinations performed, outcome and length of treatment and need for surgical interventions was retrieved from the medical records and further analyzed with statistical methods. What did the researchers find? Of all 322 patients 31% stopped the treatment within the first year, most often due to lack of stable symptom control or side effects. Less than one of 100 patients had to undergo surgery. More than half of all patients continued the treatment for at least one year and one-third for at least two years. Laboratory tests and endoscopic examinations used to assess ongoing inflammation improved. What do the findings mean? This study provides reliable information on the routine use of ustekinumab in patients with Crohn’s disease and persisting disease symptoms despite several previous treatments attempts. More than half of these difficult-to-treat patients had long-term benefit of this biologic and the drug was most often well tolerated.

## Introduction

The treatment of inflammatory bowel disease (IBD) has significantly improved with the introduction of monoclonal antibodies against tumour necrosis factor (anti-TNF) and α4β7 integrin (vedolizumab). Nevertheless, a considerable proportion of patients either fail to respond or lose response over time.^1–3^ These non-responding patients are in need of other treatment options with different modes of action, where today interleukin inhibitors (ustekinumab and risankizumab), Janus kinase inhibitors and other integrin inhibitors are available in clinical practice. Recently, emerging support to use some of these latter drugs as first-line treatments, at least in specific situations, has become available.^4^

Ustekinumab is a fully human monoclonal IgG1k antibody against the p40 subunit of interleukin 12 and 23, approved in 2016 in Europe for the treatment of moderate-to-severe Crohn’s disease (CD) following two phase III randomized controlled trials (RCTs)^5,6^ and for ulcerative colitis in 2019.^7^ In addition, the efficacy of ustekinumab has been demonstrated in long-term extensions of RCTs and in *post hoc* endoscopic outcome analysis.^8–10^

The necessary design of RCTs tend to exclude a large fraction of patients and therefore included patients often differ substantially from those in routine care.^11^ Any ‘new’ drug introduced following RCTs needs to be assessed in larger groups of unselected patients to gain a more generalizable knowledge of merits and shortfalls. As such, real-world data studies are vital to complement the experience gained from RCTs. They provide robust effectiveness data and may reveal previously undetected safety issues, both which are much needed for a reliable assessment of new therapies.

Real-world evidence on ustekinumab treatment in IBD patients receiving an intravenous induction dose followed by subcutaneously administered maintenance therapy is limited. Available studies with real-world data, mainly in CD, assess short-term effectiveness.^12–19^

The aim of this retrospective long time follow-up study was to evaluate clinical response with emphasis on steroid-free remission, need for intravenous re-induction, drug persistence, need for escalated medical treatment, hospitalization and/or surgery, to better understand the experience of ustekinumab in clinical practice.

## Methods

### Study population

All patients with CD that started treatment with ustekinumab at four major teaching hospitals in Stockholm, Sweden, from October 2016 until 31 July 2021, were included.

Inclusion criteria were age ⩾18 years and an established diagnosis of CD according to conventional clinical, endoscopic, radiological and histopathological criteria. All patients that had received at least one intravenous induction dose were included. Early patients who started with subcutaneous injections before the intravenous preparation became available, were also eligible. Exclusion criteria were concurrent participation in a clinical trial in which IBD treatment was dictated by a study protocol, prior exposure to ustekinumab or planned cessation of treatment within 12 months for any reason.

### Study design and follow-up

Retrospective multicentric study of patients followed at regular out-patient visits, in general between two to four times yearly depending on local hospital routine. The index date was the exact day of the administration of the first intravenous infusion of ustekinumab (or occasionally of the first subcutaneous injection of ustekinumab). Follow-up lasted until the end of treatment for any reason or until 31 July 2021, which came first. For patients withdrawn from treatment with ustekinumab, follow-up data including changes in medical treatment, hospitalization and surgeries were collected over the ensuing 12 months.

The reporting of this study conforms to the Strengthening the Reporting of Observational Studies in Epidemiology statement.^20^

### Data collection

Data from the onset of CD-diagnosis was retrieved from medical records in the individual hospitals’ databases using a questionnaire developed for this study, including demographics, disease characteristics, drug history before and after ustekinumab, as well as clinical follow-up data, including blood and faecal inflammatory markers, weight and endoscopic data when available. All data were entered into electronic case reporting forms and directly transferred into an anonymized electronic database using Research Electronic Data Capture technique (RedCap)^21,22^ hosted at Karolinska Institutet.

Data were collected for fixed time intervals; at baseline, which was defined as the day for administration of the first dose of ustekinumab, and at 3, 6, 9, 12, 24 and 36 months where available. Endoscopic and radiological investigations within 3 months pending baseline were considered as investigations at baseline. Observations between the fixed time points were transferred to the next scheduled time point, applying last observation carried forward-techniques. No data were collected after the preset study termination date, being 31 July 2021. When treatment was ongoing at least until 31 July 2021, data were collected at that point in time and classified as last follow-up on ustekinumab.

For descriptive statistics, the following information was collected at baseline: age, sex, smoking status, duration of disease, Montreal classification, previous medical and surgical treatment, clinical activity [Harvey Bradshaw index (HBI), physician global assessment (PGA)], biochemistry [haemoglobin, C-reactive protein (CRP), albumin, faecal calprotectin] and endoscopic activity. During follow-up, clinical and endoscopic activity, biochemistry, any change is dosing and the reason for termination of ustekinumab was collected.

### Outcome measures

Primary outcome measures were (i) clinical steroid-free response and remission at 3, 6, 12, 24 and 36 months, with main focus on 3 and 12 months and (ii) ustekinumab persistence (drug survival) at these time points.

Secondary outcome measures included (i) dose optimization (adjusting dose interval and/or intravenous reinduction with ustekinumab) at any time point caused by a change in disease activity, (ii) endoscopy results, (iii) additional medical treatments and (iv) hospitalizations and surgeries during subcutaneous maintenance treatment (beyond 8 weeks). These data were also collected during the 12-month period following ustekinumab withdrawal for any reason.

Overall disease activity was graded on a 4-grade scale as a PGA, by one senior gastroenterologist at each hospital without access to endoscopy or laboratory findings. Presence of diarrhoea, stool frequency, abdominal pain, fatigue, presence of fever and weight loss were considered and a composite overall assessment reached. Grade 0 was clinical remission; grade 1, mild disease activity; grade 2, moderate disease activity; grade 3, severe disease activity.^23^ During follow-up, patients were classified as responders (any decrease in PGA ⩾1 from the time point for the first ustekinumab dose) or as non-responders (no change or an increase in PGA). When available, HBI activity scores were collected^24^, response was defined as a decrease ⩾3 from baseline and remission as ⩽4.

All endoscopic investigations were classified as either being in endoscopic remission (grade 0) or with active disease (grade 1). In order to obtain a detailed grading of inflammation, and minimizing interobserver variability, all colonoscopies were re-evaluated by meticulous review of the endoscopy reports and available pictures by one experienced endoscopist (CH) and scored with the simple endoscopic score of CD (SES-CD).^25^ Endoscopic response was defined as reduction of the SES-CD score by ⩾50% from baseline and remission was defined as SES-CD scores between 0 and 2.^26^

For patients with ileocecal disease who had undergone an ileocecal resection, Rutgeerts’ score^26^ (0–4) was instead used. Endoscopic response was defined as a reduction of Rutgeerts’ score with at least one point. Endoscopic remission was defined as Rutgeerts’ score 0–1.^27^

Reasons for discontinuation of ustekinumab was for each patient characterized, as (i) primary non-response, PNR: absence of overall clinical improvement after intravenous initiation of ustekinumab without or with one or more subcutaneous injections, (ii) secondary loss of response, LOR: increase in disease activity after initially having obtained clinical response at any time point, (iii) adverse events or (iv) other reasons, including surgery, pregnancy and maintained stable clinical remission.

### Statistical analysis

The patient characteristics were explored using descriptive analysis and/or chi-square test when comparing groups. In general, the continuous variables/outcomes were summarized as count data, percentage, median and interquartile range (IQR) and categorical valuables/outcomes, as proportions (%).

Analysis was performed following the intention to treat principle. Discontinuation of ustekinumab for any reason was classified as treatment failure, with exception of obtained remission. Non-parametric data were explored using paired *t* test, repeated measurement one-way ANOVA, non-parametric Friedman test or Wilcoxon signed-rank test to assess changes between baseline and respective time points of follow-up. The proportion of patients with response and remission at different time points was assumed to follow a binomial distribution and 95% confidence intervals (CIs) were calculated accordingly. Group comparisons – for example, anti-TNF naïve *versus* anti-TNF experienced patients, men *versus* women – was tested with the Mann–Whitney or Kruskal–Wallis test. Differences between groups were assessed using the chi-square test for trend. In some analyses, the proportion of patients at different time points was calculated for patients with continued ustekinumab treatment using a per protocol model. Multivariate logistic regression was used to evaluate patient and disease characteristics as predictors for clinical response, clinical remission and drug persistence.

The results were stratified by the reason for initiating ustekinumab treatment as follows: (i) patients with a PNR to anti-TNF where anti-TNF was used most recently; (ii) patients intolerant to anti-TNF, with a LOR to anti-TNF, or discontinued anti-TNF for other reasons where anti-TNF was used most recently; (iii) patients intolerant to vedolizumab, with a LOR to vedolizumab, or discontinued vedolizumab of other reasons where vedolizumab was used most recently; (iv) patients treated both with anti-TNF and vedolizumab and failed these treatments for any reason; (v) patients naïve to anti-TNF and (vi) patients naïve both to anti-TNF and vedolizumab.

The alpha level in the analyses was set at 5%. All tests were two-tailed, and *p*-values of <0.05 were considered statistically significant. To avoid multiplicity problem, Bonferroni adjustments of the significance level was used. The analysis was carried out using SPSS-v 27 and/or RStudio 2023.03.0 Build 386 © 2009–2023 Posit Software, PBC, Boston MA. Furthermore, generalized estimating equation, a statistical approach to fit a marginal model for longitudinal/clustered data analysis in non-parametric data, was applied where applicable.

### Ethical approval

The study was approved by the Institutional Review Board, ‘Etikprövningsmyndigheten’, Stockholm, on 19 February 2020 (Dnr 2020-00019) and through an amendment including three additional hospitals on 16 May 2021 (Dnr 2021-00360). The requirement for informed consent to participate was waived by the Institutional Review Board.

## Results

### Study population

In total, 322 patients, 155 women (48%), with a median age of 38 (IQR: 24) at start of ustekinumab were included (Table 1). All patients had luminal disease and 72 (22%) also fistulizing disease, of which 57 (18%) had perianal CD.

**Table 1.** Demographic and baseline characteristics of 322 CD patients treated with ustekinumab.

Parameter	Category	Descriptive statistics	*N* = 322 (%)
	Gender	Female	155 (48)
		Age (years)	38 (IQR: 24.4)
		Weight (kg)	72 (IQR: 20)
		BMI (kg/m^2^)	23.9 (IQR: 5.8)
		Active smoker at baseline	36 (11)
		CD duration before ustekinumab (years)	8 (IQR: 12.5)
		Prior surgery	151 (47)
Montreal classification	Age at diagnosis	<16 years	55 (17)
		17–40 years	195 (61)
		>40 years	72 (22)
	Location	Ileal L1	48 (15)
		Colonic L2	138 (43)
		Ileocolonic L3	131 (41)
		Isolated upper L4	24 (7)
	Behaviour	B1 non-structuring/non-penetrating	183 (57)
		B2 stricturing	109 (34)
		B3 penetrating	72 (22)
		Perianal	57 (18)
	Prior medication	Infliximab	190 (59)
		Adalimumab	215 (67)
		Vedolizumab	110 (34)
		Golimumab	38 (12)
		Thiopurines	173 (54)
		Methotrexate	44 (14)
	Concomitant medication	Oral corticosteroids	(28)
		Mesalazine	27 (8)
		Thiopurine	57 (18)
		Methotrexate	4 (1)

Values are medians (IQR).

BMI, body mass index; CD, Crohn’s disease; IQR, interquartile range.

**Figure 1.**
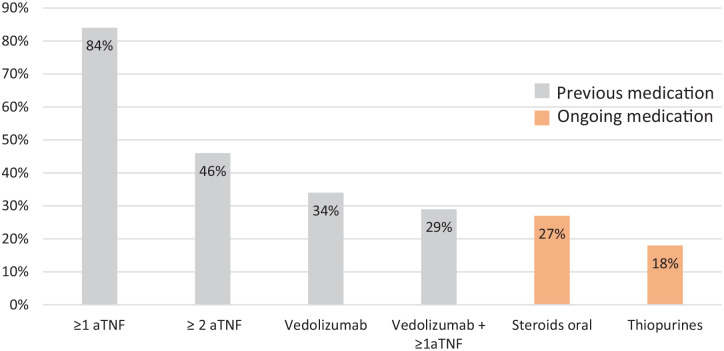
Proportion of patients previously exposed to different biological treatments and proportion of patients with ongoing immunomodulators and corticosteroids when starting ustekinumab (baseline). aTNF, anti-TNF-agents.

In total, 173 patients (54%) had previously tried and stopped thiopurines and 271 patients (84%) had failed ⩾1 and 148 (46%) ⩾2 anti-TNF drugs. Overall, 110 patients (34%) failed vedolizumab. In total, 92 patients (29%) had both failed ⩾1 anti-TNF and vedolizumab (Figure 1). Of 51 patients naïve to anti-TNFs, 18 patients had failed vedolizumab and the remaining 33 patients (10%) were naïve both to anti-TNFs and vedolizumab.

When starting ustekinumab, 299 patients (93%) had active disease and 23 (7%) were in remission. A total of 91 patients (28%) were on oral and 12 patients (4%) were on local corticosteroid treatment; 57 (18%) were on thiopurines (Table 1, Figure 1).

Of the 322 included patients, 310 patients (96%) were followed for at least 3 months and 263 patients (82%) were followed for at least 12 months; the remaining patients had their 3 and 12 months follow-up beyond the preset study termination date, 31 July 2021. The median follow-up time on ustekinumab treatment was 13.5 (IQR: 28.5) months.

**Figure 2.**
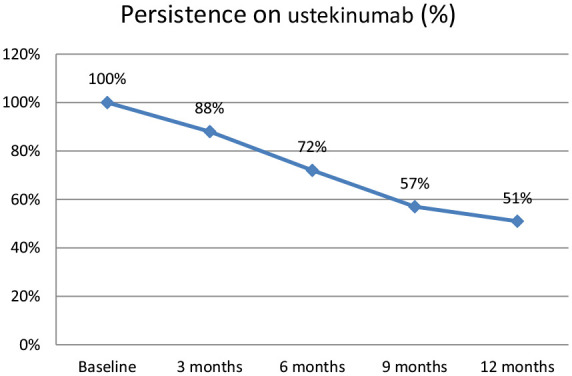
Drug persistence in 322 patients with CD during the first 12 months on ustekinumab. CD, Crohn’s disease.

Overall, the persistence on ustekinumab was 284/322 (88%) at 3 months and 165/322 (51%) at 12 months (Figure 2). Withdrawal rate was 31% (*n* = 98) during the first 12 months, mainly due to persisting disease activity (69/98, 70%) (Table 2). No observation regarding persistence at 12 months was available for 18% (*n* = 59) of patients, since the preset study termination date occurred before 12-months. A lower persistence rate was observed in anti-TNF-naïve patients only exposed to vedolizumab prior to ustekinumab [unadjusted odds ratio (OR): 0.34 (95% CI: 0.11–0.94), *p* = 0.049] compared to patients previously exposed to anti-TNF.

**Table 2.** Reasons for discontinuing ustekinumab during the first 12 months of treatment in CD.

Reasons for discontinuing ustekinumab	*N* = 98
Primary non-response	53
Loss of response	16
Adverse event/intolerance	16
Surgery	2
Pregnancy	1
Remission	1
Malignancy	1
Lost to follow-up	2
Other	6

CD, Crohn’s disease.

In total, 16% (*n* = 53) were classified as non-responders, where 8% (*n* = 27) were withdrawn after the first i.v. induction; 5% (*n* = 16) had a LOR. In addition to persisting disease activity, 16 patients (5%) were withdrawn due to adverse events. Of these, eight patients were withdrawn during the first 3 months, and the other eight patients were withdrawn between 3 and 12 months. Two patients (1%) were withdrawn due to surgery, and one patient each due to malignancy or pregnancy. Two patients were lost to follow-up; there were no deaths (Table 2).

The patient with malignancy was a 34-year-old male who underwent an emergency laparotomy due to small bowel obstruction 9 months after start of ustekinumab. At surgery, a highly differentiated small bowel adenocarcinoma with local spread was found. Following chemotherapy and a second resection, he ultimately died 2 years later. Two magnetic resonace imaging studies had been performed, one before the commencement of ustekinumab and the other after 5 months treatment. Both investigations showed ‘asymmetrical’ small bowel thickening at the same location where the adenocarcinoma later was found, a finding which had been interpreted as inflammation.

Eight-week dosing interval was the most common both at 3 months (53%) and at 12 months (52%), followed by 12-week interval which was 39% at 3 months and 33% at 12 months. During the first 12 months, a slight increase of dosing at 4- and 6-week interval was observed, from 5% at 3 months to 12% at 12 months. Intravenous reinduction was carried out in a total of six patients (1.9%) during the first 12 months.

**Figure 3.**
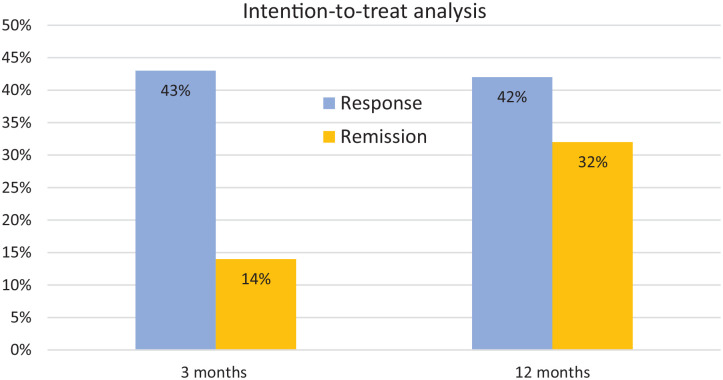
Clinical response and remission in 299 patients with active CD treated with ustekinumab. Intention-to-treat analysis. CD, Crohn’s disease.

The intention-to-treat response rate was overall 43% (130/299), at 3 months and at 12 months 42% (127/299). Of the patients with disease activity at baseline 14% (41/299) obtained remission at 3 months and 32% (96/299) at 12 months (Figure 3). Patients previously exposed to both anti-TNF and vedolizumab obtained remission [adjusted OR: 0.35 (95% CI: 0.17–0.70), *p* < 0.01] less often than patients exposed only to anti-TNF. Age, sex and other demographic variables were not related to clinical remission at 12 months.

Of the 23 patients in clinical remission at inclusion, 19/23 (83%) maintained remission at 3 months and 15/23 (65%) at 12 months. Overall, 14 (61%) were in remission at all three time points.

**Figure 4.**
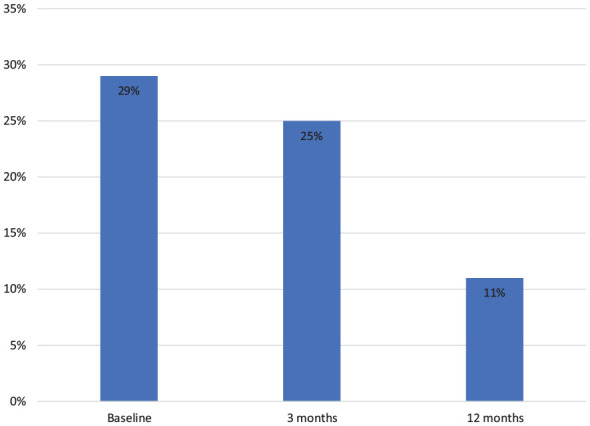
Proportion of CD patients on oral corticosteroids at baseline, and at 3 and 12 months under ongoing treatment with ustekinumab. Per-protocol analysis. CD, Crohn’s disease.

**Figure 5.**
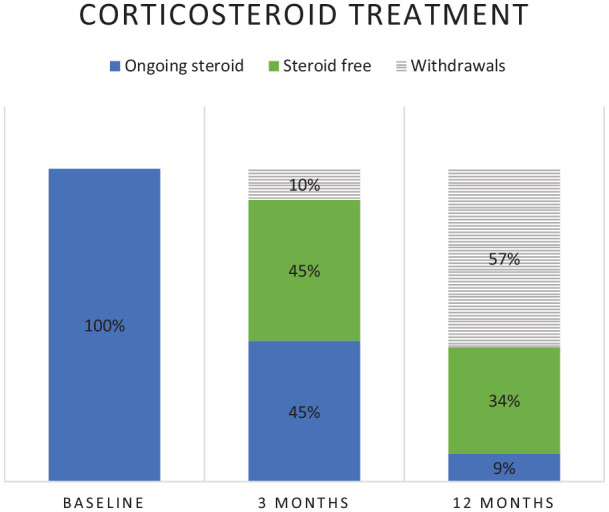
Outcome of corticosteroid treatment at 3 and 12 months in 91 CD patients with oral corticosteroids when commencing ustekinumab (baseline). Per-protocol analysis. CD, Crohn’s disease.

The proportion of patients on concomitant oral corticosteroids at baseline was 28% (91/322), at 3 months, 25% (70/284) and at 12 months, 11% (18/165) (Figure 4). Of the 91 patients initially on corticosteroids, 82 and 39 were still on ustekinumab and 50% (41/82) and 79% (31/39) were steroid-free at 3 and 12 months, respectively (Figure 5). Of the 231 patients without corticosteroids at baseline, 43 (19%) started steroids any time during the first 12 months, corresponding figures for thiopurines were 8/265 (3%).

**Figure 6.**
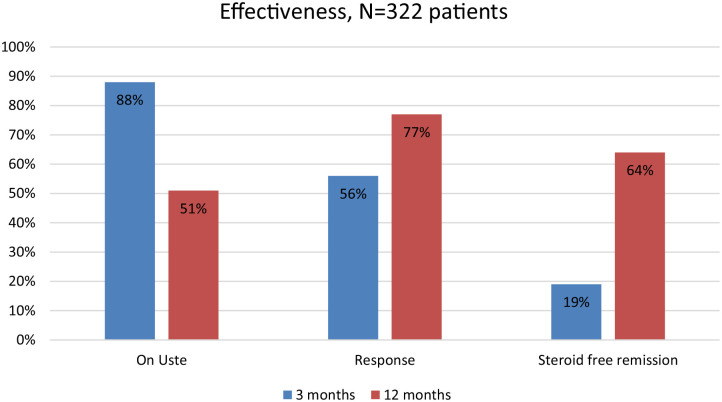
Overall drug persistence in the initially 322 patients with CD treated with ustekinumab and per-protocol analysis of clinical response and clinical steroid-free remission at 3 months (*n* = 284) and 12 months (*n* = 165). Data presented is proportion (%) of patients. CD, Crohn’s disease.

By per-protocol analysis, that is, for patients on ustekinumab, the proportion in steroid-free remission at 3 and 12 months was 19% (55/284) and 64% (105/165), respectively (Figure 6).

The median (IQR) faecal calprotectin level declined from 460 (1059) µg/g at baseline to 156 (563) µg/g at 3 months and 182 (985) µg/g at 12 months (non-significant). CRP was stable from baseline to 12 months with a median value of 4 mg/L; levels of haemoglobin (non-significant) and serum albumin (*p* < 0.05) increased slightly (Table 3).

**Table 3.** Evolution of laboratory parameters and body weight during 12 months treatment with ustekinumab in CD.

Parameter	Time at measurement			*p*-Value^a^
	Baseline *N* = 322	3 months *N* = 284	12 months *N* = 165	
	Median (IQR)	Median (IQR)	Median (IQR)	
CRP (mg/L) Measurements *N*	4 (8) 291	4 (7) 231	4 (7) 108	0.109
Haemoglobin (g/L) Measurements *N*	134 (20) 297	137 (19) 237	137 (14) 115	0.088
Albumin (g/L) Measurements *N*	37 (6) 281	38 (6) 227	38 (5) 103	0.040
Faecal calprotectin (µg/g) Measurements *N*	460 (1059) 116	156 (563) 82	182 (985) 40	0.061
Weight (kg) Measurements *N*	72 (21) 266	75 (22) 108	68 (20) 49	0.066

a *p* Values according to Friedman test.

CD, Crohn’s disease; CRP, C-reactive protein; IQR, interquartile range.

### Endoscopic evaluation

At baseline, colonoscopy or sigmoidoscopy had been performed on 179 patients (56%), of which 14 patients (8%) were in endoscopic remission and 165 patients (92%) with endoscopic inflammation. SES-CD gradings were available in a subgroup of 64 patients (20%) with a median score of 8 (IQR: 5.4); 12 patients were graded with Rutgeerts’ classification, with a median of 3 (IQR: 1.4).

At 3 months, 44 endoscopies had been performed, 14/44 = 32% were in endoscopic remission; 16 patients had SES-CD grading with a median value of 8 (IQR: 4.5). At 12 months, 34 endoscopies were performed, 15/34 (44%) were in endoscopic remission; 19 were graded with SES-CD and a median of 3 (IQR: 2.6).

Among the 165 patients with endoscopic activity at baseline, 24 patients had a follow-up investigation; at 3 months, 11/24 patients (46%) had achieved endoscopic remission; similar figures were observed at 12 months, 10/24 patients (42%). SES-CD gradings both at baseline and at 3 months were available for nine patients, the median SES-CD was 5 (IQR: 6) and 3 (IQR: 6), respectively (*p* < 0,01). Fifteen patients had a colonoscopy both at baseline and 12 months, the median SES-CD was 6 (IQR: 4) and 3 (IQR: 2), respectively (*p* < 0.01).

### Outcome beyond 12 months

#### Response and remission

At 12 months, 165 patients were still on ustekinumab; grading of disease activity was available in 160; 111 patients (69%) were in clinical remission of which 72 (65%) maintained remission until 24 months and 8 (7%) had an increase in disease activity, mostly from remission to mild activity. Of 49 patients (30%) with active disease at 12 months, 14 (29%) responded of which 9/49 patients (18%) went into remission between months 12 and 24. In total, 28/49 patients (57%) maintained the same activity grading, whereas 3/49 (6%) patients with mild disease activity at 12 months, had increased activity at 24 months. Among the 23 patients in clinical remission at inclusion, 10 (43%) maintained remission throughout 24 months; 8 of these 10 individuals had stricturing disease behaviour.

#### Persistence of ustekinumab beyond 12 months

Overall 67% of the initial population (217/322) were followed for at least 24 months.

At 12 months, 165 of the original 322 patients (51%) were still on ustekinumab treatment (Figure 2) and at 24 months, 110 patients (34%). At 24 months, in all 107 patients had been withdrawn and another 105 patients were beyond the preset study termination date (31 July 2021). At 36 months, 65 patients (20%) were still on ustekinumab and 111 patients had been withdrawn (Figure 2, Table 4). Only 25 patients had been followed beyond 36 months and were considered too few to provide meaningful information.

**Table 4.** Persistence of ustekinumab treatment in 322 patients with CD: withdrawals over time including beyond the preset study termination date.

Follow-up (*N*)	On ustekinumab	Withdrawals	Beyond preset study termination date
3 months	284	26	12
12 months	165	98	59
24 months	110	107	105
36 months	65	111	146

CD, Crohn’s disease.

#### Additional medical therapy

Of 147 patients without corticosteroids at 12 months, 6 (4%) started steroids any time between 12 and 24 months, corresponding figures for thiopurines were 1/136 (0.7%). Between 24 and 36 months, no patient started thiopurines, whereas 3 of 88 steroid-free patients needed additional steroid treatment.

#### Safety

Three patients were withdrawn beyond 12 months due to adverse events or intolerance. One patient with fistulizing disease suffered a severe perianal infection with need for surgery, one patient each had prurigo or ‘other’. No late malignancy or pregnancy occurred.

#### Hospitalizations and surgeries

Overall, seven patients (2%) were hospitalized due to worsening of disease, and eight patients (2%) were withdrawn pending bowel surgery. Four patients were hospitalized during months 12–24, and another four between months 24–36; altogether, 15 patients (5%) were hospitalized during 3 years. One patient was hospitalized both during the first and second year, and another patient hospitalized each year during the 3 years. Two patients had surgery beyond the first 12 months.

#### Follow-up during 12 months after ustekinumab withdrawal

We collected data over the 12 months following withdrawal of ustekinumab, irrespective of reason, in 121 patients. About 64% of patients (77/121) had active disease most of the time, 26% of patients (32/121) were in remission most of the time and, for 10% of patients (12/121) disease activity was unknown. Overall, 68% of patients needed additional biologic treatment, 43% of them had corticosteroids, 29% were hospitalized and 24% underwent abdominal and/or fistula surgery (Table 5). Two patients withdrew due to pregnancy or death.

**Table 5.** Medical treatment and surgeries during the 12 months after withdrawal of ustekinumab.

Treatment after withdrawal of ustekinumab	*N* = 121 (%)
Uptake new biologic	82 (68)
Vedolizumab	31 (26)
Golimumab	17 (14)
Infliximab	16 (13)
Adalimumab	10 (8)
Certolizumab	3 (3)
Other biologics	5 (4)
Other treatments	
Corticosteroids	52 (43)
Thiopurines	9 (7)
Mesalazine	6 (5)
Methotrexate	1 (1)
Tofacitinib	3 (2)
Leukapheresis	5 (4)
Other treatment	1 (1)
Surgery	
Abdominal surgery	21 (17)
Fistula surgery	9 (7)

## Discussion

This detailed retrospective study on the long-term effectiveness, including endoscopy of ustekinumab treatment in 322 patients with CD mainly refractory to treatment with anti-TNF drugs and/or integrin inhibitors, had a median follow-up on ustekinumab treatment of 13.5 months; overall 67% of patients (215/322) were followed for at least 24 months. Among withdrawn patients, data were collected over a 12-month period following the withdrawal of ustekinumab, and revealed persisting disease activity with need for additional interventions in the majority.

A number of studies have utilized real-world data to assess the long-term effectiveness and persistence of ustekinumab maintenance therapy.^28–41^ However, real-world data of objectively assessed disease activity, such as faecal markers and endoscopic outcomes, are limited.^10,18,34,35^

The ustekinumab persistence rate was high at 3 months (88%) and comparable to previous reports (90–92%^18,35^). Approximately half of the patients (51%) were still on drug at 12 months, a proportion slightly lower than in previous reports (64–83%).^18,34,35,42,43^ The lower persistence rates observed at 12 months in this study may be attributed to 18% (59/322) of patients having their 12-month follow-up beyond the preset study termination date. Nevertheless, it is probable that some of these patients continued ustekinumab treatment for at least 12 months, indicating persistence rates likely to exceed 51%. In addition, the main reason for withdrawal from maintenance therapy was persisting disease activity, reflecting a high disease burden in the patient cohort with already previous unresponsiveness to treatments, including several biologics.

The short-term (3 months) response and remission rates on treatment, 43% and 14%, respectively, were comparable to previous reports^33^ and slightly higher than in a previous Swedish study,^35^ despite similar ustekinumab retention persistence (88% and 92%, respectively). In per-protocol analyses at 12 months, the proportion of patients in steroid-free remission (64%) parallels or exceeds those previously reported, 32–53%.^33,34,36^ Response rates were dependent on previous biological exposure; only one of four patients that had failed both anti-TNF therapy and vedolizumab responded to ustekinumab. This underlines that the benefit of ustekinumab is higher in patients not previously exposed to advanced therapies^5,6^; still the overall response rate was satisfactory in already biologic-experienced patients. The refractory nature of our cohort is further underlined by the outcome during the 12 months following withdrawal of ustekinumab; additional corticosteroids were administered to almost half (43%), biologics to 7 of 10 patients (68%), and a quarter (29%) was hospitalized or underwent surgery (24%) (Table 5).

The systemic inflammatory markers were in a large proportion of our patients comparable to normal at baseline and unchanged during treatment. Early in the treatment course, faecal calprotectin levels were reduced to levels consistent with clinical remission (<250 mg/L) (Table 3).

Endoscopic evaluation at baseline had been performed in a subgroup of patients (56%). In those with endoscopic activity and a follow-up examination at 3 months, 46% achieved endoscopic remission, the proportion was the same at 12 months. Overall, endoscopic improvement was present in almost half (42%) who had a follow-up investigation within 12 months. In a small group (*n* = 15) of patients who had colonoscopy both at baseline and at 12 months, there was a significant decrease in SES-CD.

Of patients with persistent active disease at 12 months, approximately one out of three (29%) had a clinical response later while more than half (57%) maintained the same disease activity. It is therefore reasonable to consider that patients with ongoing active disease after 12 months of ustekinumab treatment, need to be re-evaluated if to continue ustekinumab or be changed to another therapy.

The treatment with ustekinumab was overall well tolerated. Withdrawal in the first year was in most patients (21%) due to persisting disease activity. Like prior studies in Swedish and Dutch populations,^35,36^ 5% of patients withdrew due to adverse events, and the remaining withdrawals were due to other reasons, such as small numbers for surgery and pregnancy. One probably unrelated small bowel carcinoma was encountered where magnetic resonace imaging-findings prior to ustekinumab had been interpreted as being of inflammatory origin. Recent studies on European populations have shown similar adverse event rates (7%), whereas cohorts in Asia had much higher adverse event rates (17–41%).^37,39^ In line with early withdrawal rates, few events were observed after 12 months; three patients were withdrawn due to adverse events of which one was due to severe infection.

Although treatment intensification occurred during the first 12 months, with a proportionally increasing number of patients receiving 6- and 4-week dosing intervals, the occurrence of adverse events remained unchanged between 3 and 12 months. Reinduction was administrated to few patients (2%) and occurred only during the first year of treatment. In a US study with a similar cohort, the reinduction rate was higher (12%) and took place mainly after 12 months of treatment. However, that study included reinductions which occurred after a break in therapy, whereas reinduction in our cohort was considered only during ongoing ustekinumab treatment, which may explain the difference.^42,43^

In general, dose escalation is a common strategy, in patients who experience LOR or insufficient response to ustekinumab, despite being off label. One thorough metanalysis reports 15 studies where dose escalation was undertaken in approximately 20% of patients during the first 40 weeks of treatment. The majority of patients (83%) underwent shortening of dosing interval and 12% received intravenous reinduction.^44^

Our overall results reflect the long-term treatment outcome of ustekinumab in patients with CD who failed other biologic agents. At baseline, only 10% of patients (33/322) were bio-naïve. In the first year of treatment, changes in clinical activity, withdrawals and adverse events were more common than later. This suggests that the benefits of ustekinumab treatment typically occur in the first 12 months, and also, that stringent monitoring is required mainly in the first year. Other advantages of the study are: (i) inclusion of a large group of unselected CD-patients, (ii) a follow-up period of up to 3 years and (iii) characterization of the disease evolution after withdrawal of ustekinumab.

The shortcomings of the study are those anticipated in retrospective studies; the application of established activity indices was limited to a subgroup of patients, as were stringent time points for disease evaluation and endoscopies. However, these limitations were taken into account by re-evaluation of disease activity by one senior gastroenterologist at each participating hospital, and re-evaluation of all endoscopic findings by an expert endoscopist. Anyhow, we cannot rule out a selection bias, that is, that patients with more severe disease were more likely to undergo endoscopy. What speaks against this is, however, that a similar proportion of patients were in endoscopic (8%; 14/179) and clinical (7%; 23/322) remission before starting ustekinumab.

## Conclusion

In conclusion, this retrospective follow-up study, including one of the largest unselected cohorts of ustekinumab-treated patients with CD and a long follow-up period, provides detailed and clinically relevant evidence in difficult-to-treat patients with active CD and previous failure to one or more biologics. Ustekinumab is effective in almost half of these patients, with high drug persistence at 12 and 24 months in combination with a favourable safety profile.

## Supplemental Material

sj-docx-1-tag-10.1177_17562848241242700 – Supplemental material for Long-term real-world data of ustekinumab in Crohn’s disease: the Stockholm ustekinumab study Supplemental material, sj-docx-1-tag-10.1177_17562848241242700 for Long-term real-world data of ustekinumab in Crohn’s disease: the Stockholm ustekinumab study by Francesca Bello, Samer Muhsen, Haider Sabhan, Alexandra Borin, Fredrik Johansson, Charlotte Höög, Ole Forsberg, Christina Wennerström, Charlotte Söderman, Mikael Lördal and Sven Almer in Therapeutic Advances in Gastroenterology

## Declarations

**Ethics approval and consent to participate:** The study was approved by the Institutional Review Board, ‘Etikprövningsmyndigheten’, Stockholm, on 19 February 2020 (Dnr 2020-00019) and through an amendment including three additional hospitals on 16 May 2021 (Dnr 2021-00360). The requirement for informed consent to participate was waived by the Institutional Review Board.

**Consent for publication:** Not applicable.

**Availability of data and materials:** Research materials supporting this publication are not publicly available, but a limited number of statistical tables may be accessed after reasonable motivation by contacting the corresponding author.
